# Evaluation of reducing temporal resolution on the accuracy of aortic pulse wave velocity assessment from velocity encoded MRI

**DOI:** 10.1186/1532-429X-16-S1-P167

**Published:** 2014-01-16

**Authors:** Michiel Sala, Pieter J van den Boogaard, Hildo J Lamb, Jos J Westenberg, Albert de Roos

**Affiliations:** 1 LUMC, Leiden, Netherlands

## Background

**Figure 1:**
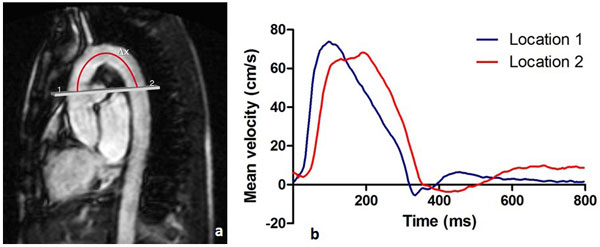
**Aortic arch pulse wave velocity assessment: (A) Double-oblique parasagittal image of the thoracic aorta**. The gray line represents the acquisition plane for one-directional through-plane velocity-encoded MRI which is positioned perpendicular to the ascending aorta (1) and additionally transects the proximal descending aorta (2). Pulse wave velocity (PWV) is determined from the propagation of the blood flow velocity, defined from the velocity-time curves recorded at locations 1 and 2 (B). PWV is defined as Δx/Δt, with Δx the path length along the centerline of the aorta from positions 1 to 2 and Δt the transit time for the foot of the velocity wave to propagate from positions 1 to 2.

Aortic pulse wave velocity (PWV), the propagation speed of blood flow velocity waves through the aorta, is a marker of aortic stiffness with prognostic value in various diseases with vascular expression. One-directional through-plane velocity-encoded (VE) MRI, planned perpendicular to the ascending aorta and additionally transecting the proximal descending aorta (Figure 1), is a validated method for assessing PWV over the aortic arch. However, the effect of the temporal resolution (Tres) of VE MRI on the accuracy of PWV assessment has not yet been established. Therefore, the aim of this study was to evaluate the effect of reducing Tres on the accuracy of aortic PWV and how this relates to physiological variation.

## Methods

Five patients referred for cardiac MRI and ten healthy volunteers within similar age range (mean age 32 ± 14 years) were prospectively included. PWV was assessed from velocity mapping using VE MRI on 3T MRI (Ingenia, Philips) with velocity sensitivity of 150 cm/s. Reference PWV (PWVref) was achieved from VE MRI with maximal number of reconstructed phases (Tres = 5 ms). The effect of temporal Tres on PWV was evaluated by temporal downsampling (50% [Tres = 10 ms], 67% [Tres = 15 ms], 75% [Tres = 20 ms], and 80% [Tres = 25 ms]), first by reducing phases during repeated reconstruction of the original acquired high-temporal data. Next, downsampling was performed by data removal as well as by data averaging from the reference PWV data set. Finally, this was compared to PWV from repeated scanning with decreasing Tres to evaluate the effect of physiologic variation in combination with temporal downsampling. Relative unsigned differences (RUDs) with respect to PWVref were calculated for downsampled data.

## Results

**Figure 2:**
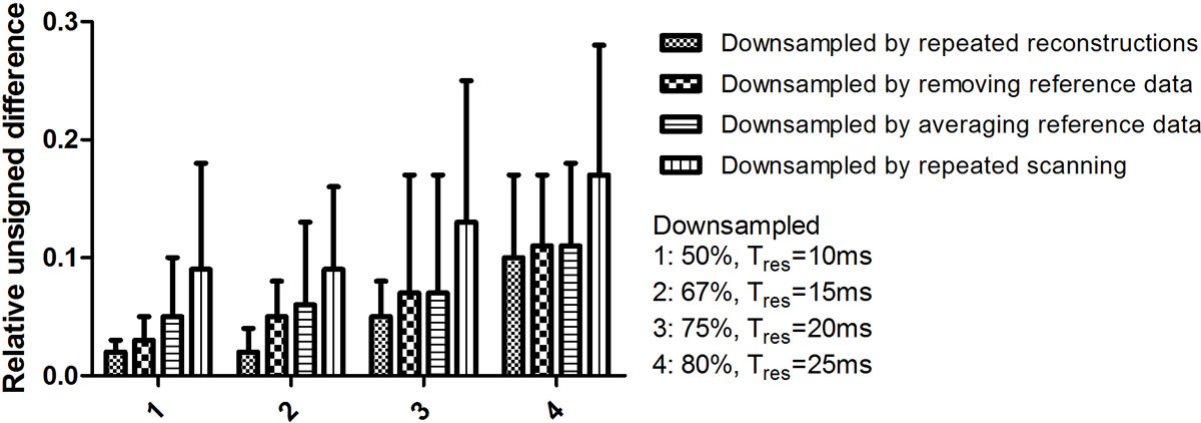
**Relative unsigned difference (RUD) in aortic arch pulse wave velocity from velocity-encoded MRI after temporal downsampling, with respect to PWV-assessment with maximal temporal resolution (PWVref)**. Downsampling (50% [Tres = 10 ms], 67% [Tres = 15 ms], 75% [Tres = 20 ms], and 80% [Tres = 25 ms]) was first achieved by repeated reconstruction of the original acquired MRI data for PWVref. Also downsampling was achieved by data removal as well as by data averaging from the reference PWV data set. Finally, downsampling was achieved by repeated scanning. Error bars represent standard deviation in RUD.

Figure 2 shows that mean RUD in PWV will increase for decreasing Tres: for Tres = 10 ms, mean RUD was 2% for downsampling by repeated reconstruction, 3% for data removal, 5% for data averaging and 9% for repeated scanning. For Tres = 25 ms, RUDs increased to 10% for repeated reconstruction, 11% for both data removal as well as data averaging and 17% for repeated scanning. Repeated scanning revealed a physiological variation in PWV of up to 7%, on top of increasing error by temporal downsampling. For repeated reconstruction, Tres of 20 ms or lower is required for RUD not exceeding 7%.

## Conclusions

Physiological changes introduce up to 7% variation in PWV. When allowing less than 7% error in PWV assessment, Tres of at least 20 ms is required. Reducing Tres in retrospective ECG-gated VE MRI does not correspond with data removal or averaging of high-temporal VE MRI data.

## Funding

Part of this research was funded by an unconditional grant provided by Dr. Rainer Bluemm.

